# Could the Epigenetics of Eosinophils in Asthma and Allergy Solve Parts of the Puzzle?

**DOI:** 10.3390/ijms22168921

**Published:** 2021-08-19

**Authors:** Émile Bélanger, Catherine Laprise

**Affiliations:** 1 Département des Sciences Fondamentales, Université du Québec à Chicoutimi, Saguenay, QC G7H 2B1, Canada; emile.belanger1@uqac.ca; 2 Centre Intersectoriel en Santé Durable, Université du Québec à Chicoutimi, Saguenay, QC G7H 2B1, Canada

**Keywords:** eosinophils, allergies, asthma, hypereosinophilia, methylation, microRNA, miRNA, epigenetics

## Abstract

Epigenetics is a field of study investigating changes in gene expression that do not alter the DNA sequence. These changes are often influenced by environmental or social factors and are reversible. Epigenetic mechanisms include DNA methylation, histone modification, and noncoding RNA. Understanding the role of these epigenetic mechanisms in human diseases provides useful information with regard to disease severity and development. Several studies have searched for the epigenetic mechanisms that regulate allergies and asthma; however, only few studies have used samples of eosinophil, a proinflammatory cell type known to be largely recruited during allergic or asthmatic inflammation. Such studies would enable us to better understand the factors that influence the massive recruitment of eosinophils during allergic and asthmatic symptoms. In this review, we sought to summarize different studies that aimed to discover differential patterns of histone modifications, DNA methylation, and noncoding RNAs in eosinophil samples of individuals with certain diseases, with a particular focus on those with asthma or allergic diseases.

## 1. Introduction

Inflammation is a central part of human immunity and helps to prevent various bacterial and viral infections. Although inflammation is useful to fight these invaders, in many diseases, chronic inflammation causes several symptoms that can be an extensive burden to those affected. For instance, in asthma, inflammation can induce bronchoconstriction, wheezing, shortness of breath, excessive mucus production, coughing, and chest tightness [1]. During inflammation, several cell types are recruited, including T lymphocytes, mast cells, macrophages, neutrophils, and eosinophils [2]. In fact, eosinophils are often described as the “central effector cell that is responsible for ongoing airway inflammation” [3]. The role of eosinophils in human diseases is discussed further in this review.

Another recent area of research interest in human diseases is epigenetics. This field focuses on the regulation of gene expression depending on factors such as the environment, social conditions, and nutrition, without changes in the DNA sequence [4]. Several epigenetic modifications are involved in human health and diseases, mainly histone modifications, DNA methylation, and noncoding RNAs, all of which are described in this review. Several studies have examined the differences between these epigenetic measures in individuals with allergies and asthma, providing useful insights into how these differential measures can influence the development or severity of these diseases or even aid in their diagnosis [5]. However, few studies have examined the influence of these modifications on eosinophils, which could prove useful for gaining a better understanding of the role of this cell type in asthma and allergic diseases. This review sought to list and summarize studies that employed eosinophil samples from individuals with various diseases to better understand the role of these cells; however, we focused mainly on studies that used samples from individuals with allergies or asthma. Specifically, the following queries were used in order to find studies of epigenetics in eosinophil samples in PubMed without regard for year of publication or disease phenotype: “(Methylation) and (Eosinophil)”, “(microRNA) and (Eosinophil)”, “(Histone modifications) and (Eosinophil)”, “(ncRNAs) and (Eosinophil)”, and “(Epigenetics) and (Eosinophil)”. Original articles that used eosinophil samples for either discovery or confirmation cohorts were retained for this review.

## 2. Eosinophils in Human Diseases

Eosinophils were first discovered by Ehrlich in 1879. In his studies, he observed an increased number of this cell type in the blood of individuals with asthma and other atopic diseases, as well as in helminthic infections [6]. In the past few years, our knowledge of eosinophils has remarkably expanded, pointing toward their more complex role instead of that of just a pro-inflammatory cell type [7]. The normal eosinophil cell count in blood usually ranges from 350 to 600 eosinophils/μL. Generally, a higher eosinophil count in the blood is considered problematic and characteristic of certain diseases. Eosinophils play a plethora of roles in human health, particularly in homeostasis and host defense. In fact, they regulate immune cell development, provide antimicrobial, antifungal, and antiviral functions, regulate glucose generation, regenerate myocytes, and regulate brown fat [8]. A recent hypothesis indicated eosinophils as “necessary, but not required” [9]. In this sense, they play a regulatory role in homeostasis, but still possess a destructive role as part of their involvement in host defense [9].

Eosinophils are often associated with various human diseases. As previously stated, they are recruited during parasitic infections and release toxic effector mediators, such as major basic proteins (MBP) [10] and reactive oxygen species [11]. Hypereosinophilia, a core feature of various eosinophil-related diseases, is defined by a blood eosinophil count of >1500 per μL. Several diseases are characterized by hypereosinophilia. Skin diseases featuring hypereosinophilia include eosinophilic cellulitis, eosinophilic fasciitis, and eosinophilic pustular folliculitis; gastrointestinal diseases include eosinophilic esophagitis, eosinophilic gastroenteritis and colitis; and respiratory diseases include eosinophilic asthma and eosinophilic pneumonia. Hypereosinophilia is also reported in certain cancers and leukemia [12]. Despite being implicated in such a wide range of diseases, this review will focus on the role of eosinophils in asthma and allergic diseases.

**Figure 1:**
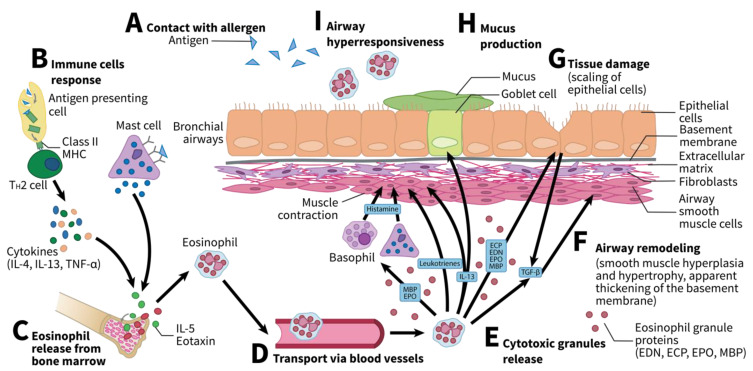
Upon allergen inhalation, several steps lead to eosinophil release. (**A**) After contact with an allergen, the antigens are absorbed by antigen presenting cells (APC) or mast cells. (**B**) APC in turn produces major histocompatibility complex II (MHC II) class molecules, allowing for T_H_2 cells to further release cytokines such as interleukin (IL)-4, IL-13, or tumor necrosis factor alpha (TNF-α). (**C**) Combined with mast cells, IL-5, and eotaxin, they promote eosinophil release from bone marrow. (**D**) Eosinophils are then transported through the vasculature to airways, where they accumulate. (**E**) They then release cytotoxic granules. (**F**) Tissue damage, as well as eosinophil recruitment, promote the release of TGF-β, which, in turn, promotes airway remodeling. (**G**) Eosinophils also promote tissue damage by releasing cytotoxic granules (eosinophil cationic proteins [ECP], eosinophil-derived neurotoxin [EDN], eosinophil peroxidase [EPO], and major basic proteins [MBP]). (**H**) They can promote excessive mucus secretion by goblet cells when releasing IL-13. (**I**) Similarly, they promote airway hyperresponsiveness either by releasing MBPs and EPO to promote the release of histamine by basophils and mast cells or directly by releasing leukotrienes or IL-13. EDN, eosinophil-derived neurotoxin; ECP, eosinophil cationic protein; EPO, eosinophil peroxidase; IL, interleukin; MBP, major basic protein; TGF-β, transforming growth factor beta; TNF-α, tumor necrosis factor alpha. Adapted from Possa et al. and McBrien et al. [14,30].

Eosinophils play an important role in asthma and allergies. Both in children and adult individuals, asthma is mainly atopic and eosinophilic. In childhood asthma, airway eosinophil numbers are usually very high, whereas blood eosinophil numbers usually remain close to normal, making it harder to use these measures as potential diagnostic tools for childhood asthma. In adult asthma, on the other hand, eosinophil numbers in airways and blood usually follow similar trends, making them a good target for potential diagnostic tools or therapies [13]. Specifically, the release of eosinophils from the bone marrow follows allergen sensitization. They then release cytotoxic granules. The pathophysiology and manifestations of asthma vary largely among individuals. However, four core features can be present: airway hyperresponsiveness (AHR), mucus hypersecretion, tissue damage, and airway remodeling [14]. Eosinophils can affect all four of these features. Briefly, the release of MBP and eosinophil peroxidase (EPO) promotes AHR either directly using leukotrienes, [15,16] or by stimulating mast cells or basophils to release histamine [17,18]. Similarly, eosinophil-derived interleukin (IL)-13 enhances the differentiation of goblet cells, promoting mucus hypersecretion [19,20]. Moreover, the four types of granules released by eosinophils (MBP, EPO, eosinophil cationic proteins [ECP], and eosinophil-derived neurotoxin [EDN]) all contribute to tissue damage due to their cytotoxic properties [21,22,23,24,25,26]. Finally, the release of transforming growth factor beta (TGF-β) by these eosinophils promotes airway remodeling, specifically by increasing fibroblasts [27,28,29]. Figure 1 shows the variety of mechanisms by which eosinophils act in asthma.

Moreover, studies have found eosinophil counts to be relevant when predicting if asthma exacerbation was to occur [31,32,33]. One treatment that was found to reduce eosinophil recruitment is the use of anti-IL-5 monoclonal antibody [34]. Despite recent studies demonstrating that anti-IL-5 treatment reduced airway eosinophil numbers without necessarily modifying the functionality of the remaining eosinophil cells [35], anti-IL-5 was used in a clinical setting to reduce asthma exacerbation [34]. In fact, Mepolizumab, the most common anti-IL-5 monoclonal antibody showed up to 43% reduction in annual exacerbation rate [36]. Other treatments, such as anti-IL-13 and anti-IL-4Rα monoclonal antibodies, were also found to reduce asthma exacerbation [37] or increase lung function [38], although increasing blood eosinophil count [39]. Finally, anti-IL-33, anti-IL-25 and anti-thymic stromal lymphopoietin (TSLP) could all be potential treatments targeting eosinophils [39]. In fact, Tezepelumab, a monoclonal antibody repressing TSLP, was found to potentially reduce airway eosinophilia in asthmatic individuals [40]. Overall, eosinophil targeting treatments are still under study, but could prove useful in preventing asthma exacerbation, especially for individuals not responding to corticosteroids.

Recently, several epigenetic mechanisms have been found to be correlated with blood and tissue eosinophilia in a variety of diseases, including allergic diseases. Epigenetics is a field of study that identifies the mechanisms regulating gene expression that do not alter the DNA sequence. Specifically, epigenetic research is interested in how the environment, social conditions, nutrition, and other external components affect genetic expression [4]. Understanding epigenetics in atopic diseases, such as atopic dermatitis, allergic rhinitis, and allergic asthma, can markedly improve our understanding of their pathogenesis and clinical manifestations. Few studies have focused on the epigenetics of eosinophils, particularly because of their small proportion in whole blood. In this review, we report recent studies on the epigenetics of eosinophils in allergic diseases and asthma.

## 3. Epigenetic Mechanisms

**Figure 2:**
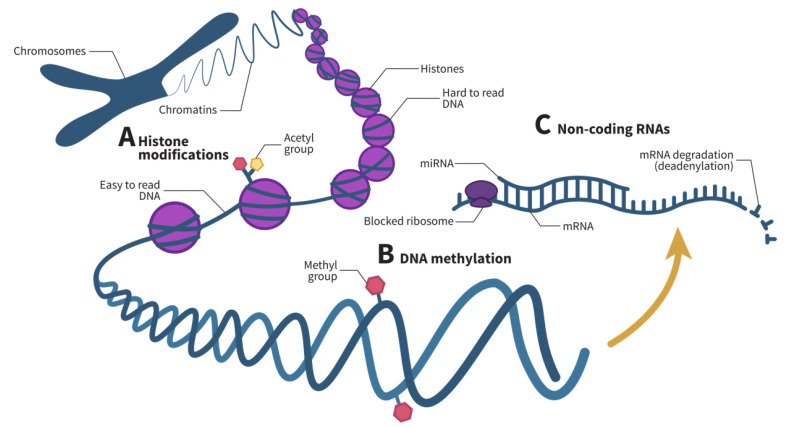
Epigenetic mechanisms act at various levels in gene expression. (**A**) Histone modifications, often including histone acetylation or methylation, untangle histones, render certain genes easier to read. (**B**) DNA methylation, on the other hand, represses gene transcription. (**C**) Finally, noncoding RNAs regulate gene expression post-transcriptionally. Specifically, microRNAs (miRNAs), one of the main noncoding RNAs that regulate gene expression, prevent messenger RNA (mRNA) translation by blocking ribosomes and speeding up mRNA degradation by deadenylation. Adapted from Chang et al. and Zhang et al. [42,43].

In the past few years, numerous epigenetic studies on human health have been reported in the literature. The environment has been found to play a role in triggering certain epigenetic mechanisms, thereby increasing or decreasing the risk of developing certain diseases. These changes are heritable and reversible [41]. Three main epigenetic mechanisms have been identified to regulate gene expression: histone modifications (Figure 2A), DNA methylation (Figure 2B), and noncoding RNAs (ncRNAs) (Figure 2C).

Histones are the basic proteins around which DNA winds to form nucleosomes. Histone modifications occur on histone tails and include acetylation, methylation, phosphorylation, ubiquitination, and a variety of other mechanisms that play a role in transcriptional regulation, DNA repair, DNA replication, alternative splicing, and chromosome condensation [44]. The two most studied histone modification types are acetylation and methylation. Overall, acetylation and deacetylation occur continuously following the movement of RNA Polymerase II, whereas methylation does not occur as dynamically as acetylation [45]. Specifically, nucleosomes are generally in a closed configuration where RNA polymerase has difficulty binding to the transcription start site. However, after enzymatic modification of histone tails, the nucleosome can unwind into an open configuration, allowing RNA polymerase to bind to the transcription start site and allow transcription [43].

DNA methylation is a process in which a methyl group is added to a DNA strand, usually in a region where a cytosine nucleotide is followed by a guanine nucleotide, commonly referred to as a CpG site, thereby silencing a gene [46]. Gene silencing can occur via a number of mechanisms. Methylated DNA can promote the recruitment of histone and chromatin modifying complexes [46,47] or inhibit transcription by repressing the recruitment of DNA-binding proteins [48]. Recently, DNA methylation has been associated with the pathogenesis of a number of immune system disorders, as well as with the differentiation, maturation, and activation of various immune cell types, including granulocytes such as eosinophils [49].

Finally, ncRNAs are RNAs that do not encode proteins. These RNAs can regulate both cell function and gene expression [50] and can be divided into two main categories: housekeeping ncRNAs, which regulate generic regular functions, and regulatory ncRNAs, which are epigenetic regulators of gene expression [50]. Table 1 presents these regulatory ncRNAs and their functions. 

microRNAs (miRNAs) are the most common regulatory ncRNAs. These short RNAs form an RNA-induced silencing complex (RISC) that binds to a target messenger RNA (mRNA) and represses its translation by blocking ribosomes and accelerating their degradation by deadenylation [51]. In the past few years, miRNAs have been studied in various diseases and cell types. In fact, some studies hypothesized that they could be used as biomarkers [57] or as potential therapeutic targets for some diseases [58].

## 4. Methylation Profile of Eosinophils

Methylation has sparked interest in the past few years as a possible regulator of various diseases. For instance, the differential methylation profiles of whole blood samples of asthmatic individuals have revealed an association with genes implicated in immune function [59]. A number of eosinophil-associated genes, namely *IL5RA* [60] and *IL13* [61], were also identified as differentially methylated in blood samples from asthmatic individuals. Other differential methylation patterns have been correlated with eosinophil cell counts in the blood [62,63]. These studies provide valuable insights into how DNA methylation can influence the development and manifestations of asthma and other eosinophil-related diseases. However, Adalsteinsson et al. found that cell-type heterogeneity in white blood cells (differences in neutrophil, lymphocyte, eosinophil, and basophil proportions) could account for up to 40% of the differences in methylation profiles of individuals [64]. It thus seems difficult to conclude that the differential methylation profiles are indeed explained by the phenotypic characteristics studied rather than by cellular heterogeneity.

An approach to overcome this limitation is to correct methylation profiles for different cell counts, as performed in previous studies. However, the best strategy is to analyze the methylation profiles of specific cell types. Few studies have identified differentially methylated CpGs in eosinophil samples. In fact, several studies found differential methylation patterns from whole blood in asthma and allergies and then replicated some of these with eosinophil methylation data available from the Saguenay–Lac-Saint-Jean (SLSJ) asthma familial cohort.

Liang et al. found that a number of differentially methylated CpGs were associated with serum IgE concentrations. Overall, methylation levels were lower in subjects with asthma combined with high IgE levels, and higher in nonasthmatic subjects. Methylation levels for asthmatic individuals with low IgE levels were between the two. A validation was performed using eosinophil data from the SLSJ cohort, confirming these findings. In fact, IgE levels were reported to be concomitant with the proportion of eosinophils in the blood and their activation in allergic diseases [65]. This study highlighted how IgE production can be regulated not only by B and T cells, but also by eosinophils, and demonstrates how studying methylation levels in a specific cell type known for its implication in the disease can provide useful information regarding the critical parameters of this disease.

Reese et al. performed a prospective analysis across eight cohorts and a cross-sectional meta-analysis across nine cohorts. The prospective analysis revealed nine CpGs and 35 differentially methylated regions (DMRs), whereas the meta-analysis revealed 179 CpGs and 36 DMRs. The researchers proceeded to validate these findings in nasal respiratory epithelium and eosinophil samples to determine if the 179 CpGs found were replicated in these cells compared to whole blood. Overall, 20 CpGs were replicated in the nasal respiratory epithelium of a Dutch cohort including 455 children, 37 of which had asthma. They also found that 128 CpGs were replicated in an African American cohort containing 36 persistent asthmatic atopic children and 36 asthmatic nonatopic children. Interestingly, 48 CpGs were replicated in eosinophil samples from 16 asthmatic children and eight nonasthmatic children in the SLSJ cohort [66].

Similarly, an epigenome-wide meta-analysis by Xu et al. revealed 14 CpGs differentially methylated between 210 asthma cases and 610 nonasthmatic patients from European cohorts. These 14 CpGs were found to have lower methylation levels in asthmatic individuals than nonasthmatic individuals. These lower methylation levels were also explained by the lower eosinophil cell counts found in the blood. A replication in eosinophil samples from the same 24 individuals of the SLSJ cohort, as previously stated, confirmed that these 14 CpGs had lower methylation levels in asthma, attributable not only to lower eosinophil blood counts but also to the lower methylation levels in these eosinophil samples [59].

Another epigenome-wide association study (EWAS) by Xu et al., including 1457 individuals in the discovery group and 1436 individuals in the replication group, found 80 candidate CpGs in childhood asthma, 21 of which were replicated in the latter. Validation with eosinophil samples from the same 24 individuals in the SLSJ cohort, as previously stated, was then performed. All 21 CpGs were found to be associated with asthma in these samples; the participants with asthma displayed on average 19% lower DNA methylation at these sites than healthy participants [67].

Over the years, the 17q12-21 locus has been most associated with asthma [68]. Madore et al. used sequencing data pertaining to locus 17q12-21 from 170 naïve CD4+ T cell samples and 145 eosinophil samples that they combined with 20 asthma-associated variants, allowing them to define expression quantitative trait loci (eQTLs) and methylation quantitative trait loci (mQTLs). These researchers found eQTLs for locus 17q12-21 associated with ORMDL sphingolipid biosynthesis regulator 3 (*ORMDL3*) and gasdermin B (*GSDMB*) genes in eosinophils, but no mQTL associations for this cell type. Finally, they found distinct methylation, eQTL and mQTL profiles between eosinophils and CD4+ T cells, further aiding in the understanding of the role of methylation of this locus in specific cell types largely implicated in the pathophysiology and clinical manifestations of the disease [69].

Finally, understanding the differential methylation profiles in eosinophils can also markedly expand our knowledge of how this epigenetic mechanism can regulate the transcription of certain genes. In fact, a study by Uhm et al. sought to understand whether the regulation, by GATA-1, of C-C motif chemokine receptor 3 (*CCR3*), a gene known to regulate eosinophil recruitment into tissues largely associated with allergies and asthma [70], could be explained by DNA methylation in eosinophil samples. Because *CCR3* transcription is increased if GATA-1 elements are unable to bind to negatively acting GATA elements, they hypothesized that DNA methylation at two CpGs located on these negatively acting GATA elements would increase the action of positively acting GATA elements. After this increase, GATA-1 would be prevented from binding to negatively acting GATA elements, effectively increasing *CCR3* transcription. To confirm this hypothesis, the researchers measured methylation in peripheral blood eosinophils, cord blood-derived eosinophils, and peripheral blood mononuclear cells from commercial Korean cell lines, which confirmed that differential methylation levels at these two CpGs increased *CCR3* transcription [71]. Table 2 summarizes these studies, all of which used eosinophils to assess the differential methylation profiles, either in their discovery or validation cohorts.

## 5. microRNA Profile of Eosinophils

miRNAs represent another epigenetic regulatory mechanism that has proven useful in our understanding of various diseases. miRNAs have been associated with eosinophil cell counts in a number of studies, usually in allergic diseases and asthma, but also in eosinophil esophagitis. However, similar to methylation studies, cell-type heterogeneity in white blood cells could partially explain the differential miRNA patterns found between individuals affected by certain diseases and healthy individuals. Although, to our knowledge, no studies have analyzed the implication of this heterogeneity in the differential miRNA patterns, the hypothesis stated by Adalsteinsson et al. with regards to methylation levels seems plausible for miRNAs.

Few studies have analyzed the differential patterns in the miRNA expression of eosinophils. One of these studies by Rodrigo-Muñoz et al. identified 14 miRNAs as potential biomarkers for asthma diagnosis. Briefly, 173 asthmatic and 53 healthy subjects were recruited, and eosinophil samples from six asthmatic individuals and four healthy individuals were retrieved for next-generation sequencing to identify differential miRNA patterns. These researchers identified 24 differential miRNAs, 14 of which were upregulated in asthmatic eosinophil samples, and the remaining 10 were downregulated. These findings were validated by qRT-PCR using the eosinophil samples of 29 asthmatic individuals and 10 healthy individuals from another group, enabling confirmation of the differential profiles of 14 miRNAs. Finally, these researchers determined whether these miRNAs could be used as potential biomarkers for the diagnosis of asthma. Briefly, they verified whether these miRNAs were conserved in the sera of 138 asthmatic individuals and 38 healthy individuals, five of which were. They then used the area under the curve (AUC) of a receiver operating characteristic (ROC) curve for the five remaining miRNAs. Based on their findings, three of these miRNAs reached a threshold of 0.7 and were thus potential biomarkers of asthma. The best potential biomarker was miR-185-5p with an AUC of 0.78, which is markedly higher than that of the previously described biomarker, periostin, with an AUC of 0.56 [72].

Similarly, Bélanger et al. isolated eosinophils from 215 individuals with or without atopic march diseases (atopic dermatitis, allergic rhinitis, asthma, or overlapping conditions). They then performed RNA sequencing on 145 eosinophil samples to find differential miRNA patterns between affected and unaffected individuals, as well as related phenotypic information such as blood and respiratory measures. These researchers found 18 differentially expressed miRNAs in various phenotypes, five of which were previously identified in asthma; 10 were downregulated in asthmatic individuals, one was upregulated in atopic dermatitis, three were positively correlated with IgE levels, and four were positively correlated with methacholine PC_20_. An analysis of the PANTHER pathways allowed them to determine the implications of these miRNAs in various pathways, notably immune response, smooth muscle cell proliferation, relaxation and growth, and tissue remodeling [73].

Allantaz et al. searched for differential miRNA patterns in immune cells, namely neutrophils, eosinophils, monocytes, B cells, natural killer cells, CD4 T cells, CD8 T cells, myeloid dendritic cells, and plasmacytoid dendritic cells. Their intent was to determine whether these immune cell-type miRNAs could regulate the expression of their target genes. Specifically, for eosinophils, miR-935 was expressed only in eosinophils, whereas miR-223 and miR-652 were expressed in eosinophils, monocytes, and neutrophils. They also identified 15 other miRNAs that were specifically expressed in one, two, or three of the aforementioned cell types. To verify whether these miRNAs effectively negatively regulated target mRNAs, they applied a seed site matching algorithm to the miRNA and mRNA probes, as well as on targets found on TargetScan. This allowed the identification of overlapping targets and negative correlations between miRNAs and mRNAs. miR-223 and miR-652 were found to be negatively correlated with a significant number of targets, two of which were specific to eosinophils, monocytes, and neutrophils [74].

Yu et al. sought to investigate the regulatory role of miR-663 in nasal polyposis in children by investigating the potential regulation of TGF-β1 by this miRNA. TGF-β1 plays an important role in fibroblast proliferation and the formation of granulation tissue, two key features of nasal polyposis pathogenesis. They extracted nasal polyp tissues or inferior nasal concha, serum, and peripheral blood eosinophils of 35 affected children and 46 unaffected children who had undergone surgical removal of the inferior nasal concha due to simple inferior turbinate hypertrophy. They measured the differential miR-663 and TGF-β1 mRNA expression between individuals by qRT-PCR and assessed the expression levels of TGF-β1 by using Western blotting. Their findings revealed that miR-663 was downregulated in eosinophil samples of individuals with nasal polyposis, while both TGF-β1 mRNA and protein were upregulated, suggesting a potential regulatory role of this miRNA in TGF-β1. Finally, these researchers determined whether TGF-β1 was a target of miR-663 by using a dual-luciferase reporter assay. They confirmed that miR-663 could bind to the 3’-UTR of TGF-β1, thereby potentially regulating its expression [75].

Finally, a study by Wong et al. investigated the potential role of miR-21*, a complementary miRNA of miR-21, on eosinophil cell survival by targeting the granulocyte-macrophage colony-stimulating factor (GM-CSF). Briefly, they isolated eosinophils from a nonallergic buffy coat. Thereafter, they transfected pre-miR-21* and found that it could upregulate miR-21* expression, enhance the GM-CSF-activated extracellular signal-regulated kinase (ERK) pathway, and potentially reverse the apoptosis of eosinophils. Therefore, this study demonstrated the potential of employing miR-21* in future research on therapeutic strategies to prevent allergic diseases [76]. Table 3 summarizes these studies.

## 6. Other Epigenetic Mechanisms of Eosinophils

Although more epigenetic modifications have been documented in human diseases, such as histone modifications and other noncoding RNAs, no studies have investigated their effects on phenotypic traits from isolated eosinophil samples. Few studies have examined the correlation between these measures and eosinophil blood counts. In a study by Grausenburger et al., the deletion of histone deacetylase 1 was found to increase critical airway inflammation parameters in a murine Th2 asthma model, including eosinophil recruitment [77].

Only one study analyzed the effect of histone modification on diverse cell types. Koyanagi used histone modification data from erythroblasts, megakaryocytes, eosinophils, neutrophils, monocytes, natural killer cells, T cells, and B cells to infer the changes that occurred during the differentiation of these cell types. This study allowed them to infer that there are several histone modification sites during hematopoiesis of these cell types, thereby improving our understanding of the genes involved in each cell-type differentiation [78].

Similarly, few studies have examined the correlations between eosinophil blood counts and other ncRNAs in whole blood. One study found a few circRNA-miRNA-mRNA triads associated with eosinophils. In particular, the researchers identified circ_0022343-miR-503-5p-solute carrier family 2 member 3 (SLC2A3) for the potential regulation of eosinophils in chronic thromboembolic pulmonary hypertension [79]. Another study found a positive correlation between circ_0005519 expression in the CD4+ T cells of asthmatic individuals and eosinophil blood counts. They suggested that circ_0005519 might bind to miRNA let-7a-5p, which is known to suppress IL-13 and IL-6 in CD4+ T cells [80]. Other studies investigated the effect of RNA interference treatments using siRNAs in allergic diseases and asthma, and associated gene silencing with reduced eosinophil infiltration [81,82,83,84,85,86,87,88,89]. These studies all shared a similar methodology in which a murine model of asthma was transfected with a specific siRNA to silence a gene known to promote inflammation, proving the potential role of these small ncRNAs in allergic diseases. However, no studies of ncRNAs, besides those mentioned in the miRNA section, appeared to isolate eosinophils to measure differential ncRNA patterns between phenotypic traits, even though cell-type variability could potentially account for a portion of the variation in ncRNA profiles.

## 7. Conclusions

Eosinophils are a central component of inflammation in human diseases and infections. As previously stated, they are recruited in allergic diseases and asthma where they play a role in AHR, mucus production, tissue damage, and airway remodeling. Although their role in these diseases remains uncertain, these pro-inflammatory cells are one of the most implicated cell-types in asthma and allergic diseases. Epigenetics is a growing field of study regarding human disease pathophysiology and manifestations. Certain diseases are complex traits, which indicates that their appearance depends on multiple factors, including genetics and epigenetics. Examples of these diseases include asthma and allergic diseases. In these diseases, it is difficult to identify a single cause. Instead, a combination of genetic predisposing factors and environmental factors is often found. It is therefore critical to assess the epigenetic mechanisms regulating the appearance or severity of allergic diseases. Several studies have analyzed differential methylation or miRNA profiles in asthma and allergic diseases, and a number of these studies have associated these epigenetic measures with eosinophil proliferation. Cell-type variability may account for these differences in epigenetic measures. A feasible strategy for determining whether the difference in these measures is caused by cell-type variability is to correct for the various cell types. However, the best approach for discovering the implication of epigenetics in a disease on a specific cell type is still to isolate the cell and then directly measure either the methylation profiles, ncRNAs, or histone modifications in the cell type.

This review sought to document studies conducted to date on eosinophil samples. These studies provide valuable insights into the post-transcriptional regulation of various diseases, including allergic diseases and asthma.

Unfortunately, only few studies have used eosinophil samples to measure critical epigenetic parameters. However, this methodology seems promising for determining the implications of each cell type on a disease to better understand the pathophysiology and clinical manifestations of these diseases, as well as to identify potential therapeutic targets or prevent these diseases to a certain extent. In fact, it would be particularly interesting to further investigate the regulatory role of circRNAs on miRNAs, which in turn influence mRNA expression in eosinophil samples of individuals with these diseases.

## Figures and Tables

**Table 1:** Regulatory ncRNAs and their targets. Adapted from Zhang et al. [50].

Abbreviation	Full Name	Size	Function	Reference
miRNA	microRNA	21–23 nt	Represses target mRNA by accelerating deadenylation and blocking ribosome	[51]
siRNA	Small interfering RNA	20–25 nt	Degrades target mRNA after its transcription	[52]
piRNA	Piwi-interacting RNA	26–32 nt	Binds to piwi proteins to silence transposons	[53]
eRNA	Enhancer RNA	50–2000 nt	Influences gene expression by modulating chromatin structure	[54]
lncRNA	Long noncoding RNA	>200 nt	Induces changes in chromatin structure	[53]
circRNA	Circular RNA	100–10,000 nt	Regulates gene expression by binding to miRNAs, preventing them from binding to mRNA	[55]
Y RNA	Y RNA	80–120 nt	Inhibits DNA replication	[56]

**Table 2:** Methylation studies of eosinophils.

Total Samples *n*	Eosinophil Samples *n*	Mechanism	Disease	Country of Eosinophil Sample Collection	Author, PMID
664	24	Methylation	Asthma	Canada	Liang et al., 25707804
1850	24	Methylation	Childhood asthma	Canada	Reese et al., 30579849
6539	24	Methylation	Childhood asthma	Canada	Xu et al., 2018, 29496485
5826	24	Methylation	Childhood allergy	Canada	Xu et al., 2020, 33338541
315	140	Methylation	Allergic asthma	Canada	Madore et al., 32312674
N/A	N/A	Methylation	N/A	Korea (commercial cell lines)	Uhm et al., 22217447

These articles were found using the following query in PubMed: (Methylation) and (Eosinophil). All articles that used eosinophil samples to measure differential methylation levels in either the discovery or validation study were retained for this review.

**Table 3:** miRNA studies of eosinophils.

Eosinophil Samples *n*	miRNAs Studied *n*	Mechanism	Disease	Country of Eosinophil Sample Collection	Author, PMID
44	14	miRNA	Asthma	Spain	Rodrigo-Muñoz et al., 30040124
145	18	miRNA	Atopic march	Canada	Bélanger et al., 33260893
N/A	18	miRNA	N/A	France	Allantaz et al., 22276136
81	1	miRNA	Nasal polyposis	China	Yu et al., 29849780
N/A	1	miRNA	N/A; eosinophil survival	China	Wong et al., 22698984

These articles were found using the following query in PubMed: (microRNA) AND (Eosinophil). All articles that used eosinophil samples to measure differential miRNA patterns in either the discovery or validation study were retained for this review.
